# Global optimization of metasurface designs using statistical learning methods

**DOI:** 10.1038/s41598-019-53878-9

**Published:** 2019-11-29

**Authors:** Mahmoud M. R. Elsawy, Stéphane Lanteri, Régis Duvigneau, Gauthier Brière, Mohamed Sabry Mohamed, Patrice Genevet

**Affiliations:** 1 Université Côte d’Azur, Inria, CNRS, LJAD, 06902 Sophia Antipolis Cedex, France; 2 CNRS, CRHEA, Université Côte d’Azur, rue Bernard Gregory, 06560, Sophia Antipolis, Valbonne France

**Keywords:** Metamaterials, Software, Sub-wavelength optics, Statistics

## Abstract

Optimization of the performance of flat optical components, also dubbed *metasurfaces*, is a crucial step towards their implementation in realistic optical systems. Yet, most of the design techniques, which rely on large parameter search to calculate the optical scattering response of elementary building blocks, do not account for near-field interactions that strongly influence the device performance. In this work, we exploit two advanced optimization techniques based on statistical learning and evolutionary strategies together with a fullwave high order Discontinuous Galerkin Time-Domain (DGTD) solver to optimize phase gradient metasurfaces. We first review the main features of these optimization techniques and then show that they can outperform most of the available designs proposed in the literature. Statistical learning is particularly interesting for optimizing complex problems containing several global minima/maxima. We then demonstrate optimal designs for GaN semiconductor phase gradient metasurfaces operating at visible wavelengths. Our numerical results reveal that rectangular and cylindrical nanopillar arrays can achieve more than respectively 88% and 85% of diffraction efficiency for TM polarization and both TM and TE polarization respectively, using only 150 fullwave simulations. To the best of our knowledge, this is the highest blazed diffraction efficiency reported so far at visible wavelength using such metasurface architectures.

## Introduction

Metasurfaces have been studied extensively in the past few years due to their exceptional abilities in achieving arbitrary light control in a very short propagation distance, and due to their simplified fabrication procedures with respect to bulk metamaterials^1–5^. Metasurfaces consist of assemblies of nanoresonators with spatially varying geometrical parameters and separated by subwavelength distances, made of plasmonic^6^ and/or high dielectric refractive index materials^5,7^. Unlike the conventional optical components that provide a full control of the light properties over long propagation distances, metasurfaces can introduce highly resolved phase, amplitude, and polarization changes on the incoming wavefront over very short propagation distances, typically in the order of the wavelength^3–8^. Owing to the versatility and the capabilities of metasurfaces, many exotic and peculiar optical phenomena ranging from negative refraction^9^, sub-diffraction optical microscopy^10^, and broadband achromatic lenses^11,12^ have been demonstrated recently using ultrathin and compact devices. Most of these designs have been engineered by considering a brute force approach. The latter consists in performing an extensive and costly parametric search to obtain the optical response of individual building blocks. Although simulations are generally performed considering array of nanostructures, this direct approach does not properly consider potential coupling effects between neighboring elements having different shapes. Remarkably, complex designs such as broadband and multiplexed interfaces exclusively rely on sub-units of near-field coupled antennas to achieve the required scattering responses.The inherent complexity of the latter designs result in poorly efficient components, indicating that direct modelling approaches are becoming substantially insufficient and are failing to achieve designs of realistic devices^13,14^. New and advanced methods, such as inverse design techniques, are becoming mandatory to further exploit metasurface capabilities in highly demanding applications^14,15^. To this end, several optimization methodologies have been developed and demonstrated in the recent years, including local and global search methods. The former is suitable to rapidly convergence to local maxima/minima and thus strongly depends on the initial parameter guess^16,17^. This category includes topology optimization^18–22^ and so-called *objective-first* algorithms^23–25^.

The second approach, performing global parameter optimization includes stochastic search techniques such as genetic algorithms^26–28^ and evolutionary algorithms^29,30^. These are general methods which are very efficient for large parameter space optimization. The downside of the latter methods is that they all require a large number of forward solver calls and are thus impractical when combined with costly (three-dimensional (3D) time-domain simulations.

In the last two years, artificial neural networks have been used to develop innovative modelling strategies for several nanoscale light-matter interaction problems including light scattering problems from spherical nanoshells for example^31^. Artificial neural networks have also been utilized recently to design efficient metasurfaces^32,33^. As a general rule, training an artificial network requires numerous training data before it becomes capable of achieving the optimized design based on a specific input target. Thus, efficient neural networks, capable of generating practical designs, require thousands of training data using a fullwave electromagnetic solver^34,35^. The computation cost could become important, especially considering 3D complex problems. Another common problem of neural networks arises when the system under investigation has many diverse parameters, i.e. when several parameter sets could give approximately the same response. In this case, the performance of the network reduces dramatically^33,34^.

The main goal of our work is to introduce to the nanophotonics community novel and significantly more advanced evolutionary optimization strategies based on derandomized and statistical learning, evolution strategies. Using practical designs of semiconductor GaN phase gradient metasurfaces, we demonstrate that these techniques can outperform most of optimization techniques used in the inverse design of metasurfaces. These are especially useful when one is considering complex 3D problems. We adopt a parametric shape optimization viewpoint as opposed to a topology optimization approach, enabling faster convergence to a global minima/maxima even for large parameter space with regards to our setting. In addition, as shown inhere, our methods are capable of achieving effectively different global minima/maxima for the same value of the objective function, involving different parameter values. We first consider an analytical example to help readers gaining insights into our inverse design tools. The second goal of this paper is to apply theses techniques to the case of 3D GaN phase gradient metasurfaces made of nanopillars of different shapes, targeting maximum light deflection efficiency at a wavelength of *λ* = 600 *nm*. The deliberate choice of GaN semiconductor has been made after a careful consideration of several factors such as the optical losses in the visible regime, its high refractive index in the visible regime, and the current ease of micro/nanofabrication technology in the industry of this materials, yielding ideal nanoresonators (phase-shifters) for metasurface designs and fabrication^7,36^. Over the last couple of years, several example of light deflecting metasurfaces have been realized both numerically and experimentally for visible wavelength applications involving GaN^36,37^ or at near infrared^21,29,38^ using silicon or hydrogenated amorphous silicon. For the latter, the absorption losses of silicon in the visible make them less efficient than, for example, TiO_2_^19^ or c-Si^19,39^. Various metagratings have been used to demonstrate efficient light deflection at visible regime but the performance does not exceed 80%^40^. Here, we provide optimized 3D metasurface designs with record efficiency above 87%. To the best of our knowledge, this is the highest expected performance reported in the literature at visible regime for 3D gradient metasurfaces. Our global optimization techniques rely respectively on advanced evolutionary strategies and statistical learning, coupled with a high order Discontinuous Galerkin Time-Domain (DGTD) solver from the DIOGENeS software suite dedicated to computational nanophotonics^41^. We apply the optimization solution to design optimal phase gradient metasurfaces made of scatterers of different shaped, such as rectangular and cylindrical nanopillars. Our calculations target maximum diffraction efficiency (*η*(*n*, *m*), where *n*, *m* are the mode indices) at *λ* = 600 *nm*. For rectangular shaped nanopillars, we achieved more than 85% of diffraction efficiency at *λ* = 600 *nm* for TM polarized waves, while cylindrically shaped nanopillar interfaces lead to more than 85% efficiency for both TM and TE light polarization at *λ* = 600 *nm*.

## Fullwave Time-Domain Solver

In this work, we consider a general modelling approach for the numerical characterization of metasurfaces based on rectangular and cylindrical nanopillars by solving the full system of 3D time-domain Maxwell equations. The most widely used numerical method for this purpose is the Finite Difference Time-Domain (FDTD) method^42^ rooted on Yee’s scheme^43^. The FDTD method is highly popular in the computational nanophotonics community, in particular due to its simplicity and computational efficiency. It is available in several commercial software such as the Lumerical suite, which has been used here for comparison with the alternative numerical method that we consider in our study. Let us first recall that in the FDTD method, the whole computational domain is discretized using a structured (cartesian) grid, which is a strong asset from the point of view of computational efficiency. However, Yee’s scheme may suffer from serious accuracy degradation when used to model curved objects or when treating material interfaces, especially if one is interested in assessing near field effects. In general, this requires a refinement of the underlying grid, which incurs a substantial increase of the simulation time and memory footprint. An alternative approach is to resort to a numerical method capable of handling more general meshes, e.g., unstructured grids. Such a method has been proposed in the early 2000’s by a few research groups in applied mathematics community and is referred as the Discontinuous Galerkin Time-Domain (DGTD) method^44^. This method can be seen as a blending of a classical (continuous) Finite Element Time-Domain (FETD) and Finite Volume Time-Domain (FVTD) method. As a consequence, the DGTD method inherits attractive features of FETD and FVTD methods and combines them to produce a fullwave time-domain solver, which is nowadays increasingly used for the simulation of electromagnetic wave propagation problems. A DGTD method relies on a high order approximation of the unknown field with a polynomial interpolation method (that we shall denote in the sequel as ${{\mathbb{P}}}_{p}$ where *p* is he interpolation degree). It is formulated on a fully unstructured tetrahedral mesh opening the route to a local refinement of the mesh and the possibility of using curvilinear cells for a high order approximation of complex geometry features. More importantly, when the discretization in space is coupled to an explicit time integration method, the DG formalism leads to a block diagonal mass matrix independently of the form of the local approximation (e.g the type of polynomial interpolation). This is a striking difference with classical, continuous FETD formulations. Finally, such a time explicit DGTD method is naturally adapted to parallel computing. In this study, we exploit a high order DGTD-based solver that we have recently developed, and which has been specifically designed for the simulation of nanoscale light-matter interaction problems^45^. This DGTD fullwave solver is implemented in the DIOGENeS^41^ software suite, which is programmed in Fortran 2008 and is adapted to high performance computing systems.

It is worth mentioning that the a metasurface device can also be modeled in the frequency-domain, especially when one is interested in assessing its performance at a few frequency points. In this context, the most widely used simulation approach is the Rigorous Coupled Wave Analysis (RCWA) method^2^. RCWA is a modal-type method. The method is based on Floquet’s theorem that the solutions of periodic differential equations can be expanded with Floquet functions. A device is divided into layers that are each uniform in the transverse direction, e.g., the *z* direction. In the horizontal plane, i.e., the plane of the metasurface layout, Maxwell’s equations (in partial differential form) are expanded by the Floquet functions and turned into infinitely large algebraic equations. With the cutting off of higher order Floquet functions, depending on the accuracy and convergence speed one needs, the infinitely large algebraic equations become finite and thus solvable by computers. Then, the electromagnetic modes in each layer are calculated and analytically propagated through the layers. The overall problem is solved by matching boundary conditions at each of the interfaces between the layers using a technique like scattering matrices. Overall, RCWA is a highly efficient method and is the method of choice for many problems involving nanostructures with simple shapes and devices with smooth variation in the *z* direction. For curved devices, a staircase approximation is needed and the RCWA can become a very expensive method as the modal discretization is refined for an improved accuracy.

## Optimization Methods

We are here leveraging on two different efficient global optimization techniques respectively based on advanced evolutionary strategies and statistical learning to perform inverse metasurface designs. The first optimization approach is the so-called “Covariance Matrix Adaptation Evolution Strategy” (CMA-ES)^46^, which belongs to the family of evolutionary algorithms. Similarly as genetic algorithms (GAs)^47^, CMA-ES mimics natural evolution principles to maximize an objective function. It has been tested on several academic benchmarks as well as industrial problems and reported as one of the most efficient optimization algorithms for a broad class of problems. Recently, it has been used to optimize infrared broadband quarter-wave and half-wave plates Bézier metasurfaces^48^, reconfigurable metasurface absorbers^49^, acoustic metamaterial^50^, and for in optimizing apochromatic singlets metasurface-augmented grin lenses^51^. This is of critical importance for the design of complex assemblies of arrays of 3D nanostructurea requiring expensive simulations with a large number of parameters, thus increasing the chance of finding several designs with relatively well-optimized performances.

**Figure 1:**
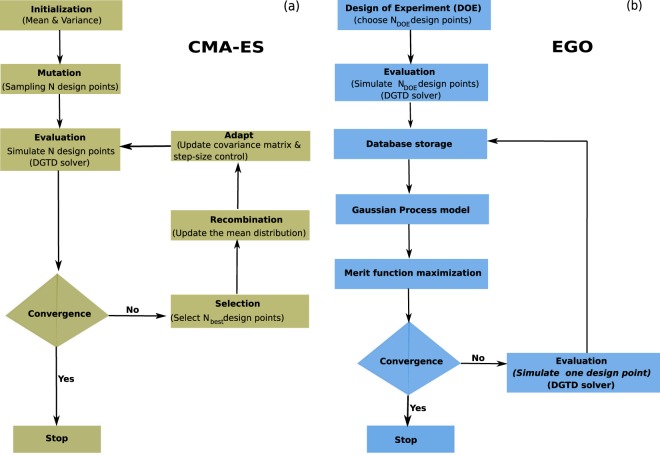
For the CMA-ES (**a**), the optimization starts with an initial design and a given mean and variance. The second step consists in generating a population of *N* designs that will be evaluated using the fullwave DGTD solver. If the convergence is not satisfied, the algorithm chooses the *N*_*best*_ designs according to the objective function, and use them to update the mean of the distribution, size and the covariance matrix. *N* new designs are thereafter calculated and repeated steps until the convergence criterion is reached. For the EGO (**b**) instead, the algorithm starts with an initial design of experiments composed of *N*_*DOE*_ designs, that will be simulated to estimate the corresponding objective function. The results are used to construct a surrogate model, of interest to search for the next design. The latter is simulated using the fullwave DGTD solver, and the corresponding objective value enrich the database, repeating these steps until the convergence is obtained. The major difference between the CMA-ES and EGO methods essentially relies on the utilization of a surrogate model for EGO that drastically reduces the number of evaluations *N*_*DOE*_ by an order of magnitude.

As other Evolution Strategies (ES), CMA-ES is based on a sequence of random searches, ruled by a normally distributed sampling. At each iteration, the characteristics of the distribution, and in particular its covariance matrix, are adapted to account for the latest obtained observations, in order to accelerate the convergence towards the maximum/minimum of the fitness function. More precisely, the algorithm starts with an initial (sometime random) distribution mean (initial design) and the identity classical normal distribution as covariance matrix scaled by a user-defined scalar variance. According to this distribution, a set of *N* samples are generated randomly (*mutation* step as shown in Fig. 1(a)) and the corresponding designs are simulated to evaluate their fitness. Then, the best *N*_best_ designs among the *N* ones are selected for the evolution (*selection step*) and are used to update the mean of the distribution (*recombination* step). Finally, the covariance matrix is updated accounting for a principal component analysis of the best points *N*_best_, whereas the scalar variance is modified using the path of the mean point (*step-size control*). The whole procedure is repeated using the updated distribution, until convergence. The detailed algorithm is described in^46,52^ and illustrated in Fig. 1(a). In the electromagnetic community, GAs are more commonly used^15,26,27,47,53^, although they suffer from some well-known drawbacks, such as the necessity to calibrate several numerical parameters and their inability to tackle anisotropic behaviors of the fitness function, i.e. the ability to change/adapt the search shape distribution during the optimization in order to tackle the complexity of the objective function for faster convergence. Consequently, GAs require a substantial amount of computational time when applied to high-performance 3D time/frequency domain electromagnetic solvers. Therefore, due to the huge number of parameters required to ensure the flexibility of modern devices, more advanced evolutionary strategies like CMA-ES are needed, that could (1) offer a faster convergence, i.e. require fewer fullwave solver runs (due to its ability to update the shape and the size of the distribution during the optimization), and (2) provide more accurate results. In Algorithm 1, we provide a simple illustration of the CMA-ES steps. For more details about CMA-ES, we refer to refs. ^46,52^.

Despite the advantages of CMA-ES over other classical evolutionary algorithms, it still requires a large number of computationally expensive simulations, which increases with the number of optimization parameters (*curse of dimensionality*).

**Algorithm 1:**
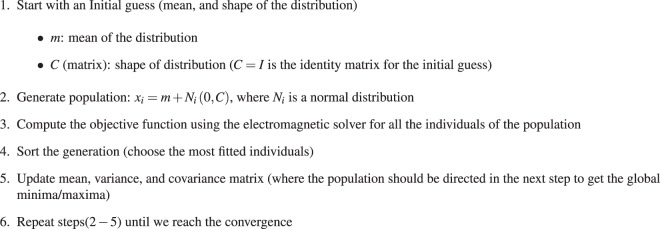
Baseline CMA-ES steps

Therefore, we also consider an alternative optimization strategy based on the iterative construction of surrogate models. It is known as Efficient Global Optimization (EGO)^54–56^ and belongs to the class of Bayesian optimization methods. Contrary to evolutionary algorithms, EGO is not based on adaptive sampling, but on a surrogate model built on the basis of available fitness observations, which is employed to decide which new design should be tested next, by maximizing a statistical criterion related to the optimization goal (*merit function*). More precisely, this approach proceeds in two main steps. First, a Design Of Experiments (DOE) is carried out, which consists in exploring randomly the admissible design space using a uniform sampling strategy (e.g. Latin Hypercube Sampling)^57^. After simulation of the corresponding designs, all fitness values obtained are considered as observations and are stored in a database. In the second step, this database is iteratively refined towards the most promising areas. This is performed using a Gaussian Process (GP) model, constructed using all the database points. This is basically an interpolating or approximating model, whose internal parameters are calibrated according to a maximum likelihood principle^58^. Once this GP model is defined, one can estimate at any point of the design space the fitness value (model mean) and an uncertainty value (model variance). Both are used to define a statistical merit function, e.g. the expected improvement, whose maximum defines the next design parameters set to evaluate. After simulation of this new point, the database is updated accounting for this new observation and the second step of the algorithm repeats until convergence. The algorithm is illustrated in Fig. 1(b). As explained, this approach based on the iterative construction of a database and an associated model can be considered as a statistical learning strategy, like for instance Artificial Neural Networks (ANNs). Its main characteristic is the use of internal uncertainty estimation (variance) to drive both the search for the optimum and the improvement of the model accuracy. Contrary to the approaches based on ANNs, that often aims at constructing a model accurate in the whole design space before optimization, EGO focuses on the most promising areas regarding the optimization criterion. It is therefore far less expensive in terms of solver calls, making it very well suited to the context of expensive simulations. In practice, only a few hundreds of simulations are typically required for EGO, whereas the database used to train ANNs usually requires tens of thousands of solver calls. As an example, in ref. ^32^ a database of 90 000 simulations has been achieved in order to train an ANN to optimize 16 parameters. For EGO, as illustrated below, we need only 80 points for the initial design of experiments (DoE) and 150 solver calls for the iterative enrichment, to optimize structures with 12 and 8 parameters (rectangular and cylindrical shaped antennas).

We would like to emphasize that EGO has some limitations in terms of the number of optimization parameters, due to the fact that it relies on the construction of an internal Gaussian Process model, to decide which new point should be evaluated. This limitation is difficult to define precisely in general, because it depends on the problem studied. In the literature, it is reported that EGO should in general not be employed above one hundred of parameters. Nevertheless, high-dimensional Bayesian optimization is a very active research topic. In particular, preconditioning methods are studied, in order to reduce the number of active parameters effectively used during optimization.

In order to highlight the different optimization behaviors and performance between CMA-ES and EGO and to give the reader more insights about their properties, we provide here a 2D analytical example which is designed to find the global minimum of a 2D Branin function, defined by:

1 $$\mathop{{\rm{minimize}}}\limits_{x,y}\,\,f(x,y)={(y-\frac{5.1}{4{\pi }^{2}}{x}^{2}+\frac{5}{\pi }x-6)}^{2}+10(1-\frac{1}{8\pi })\cos \,(x)+10,$$

**Figure 2:**
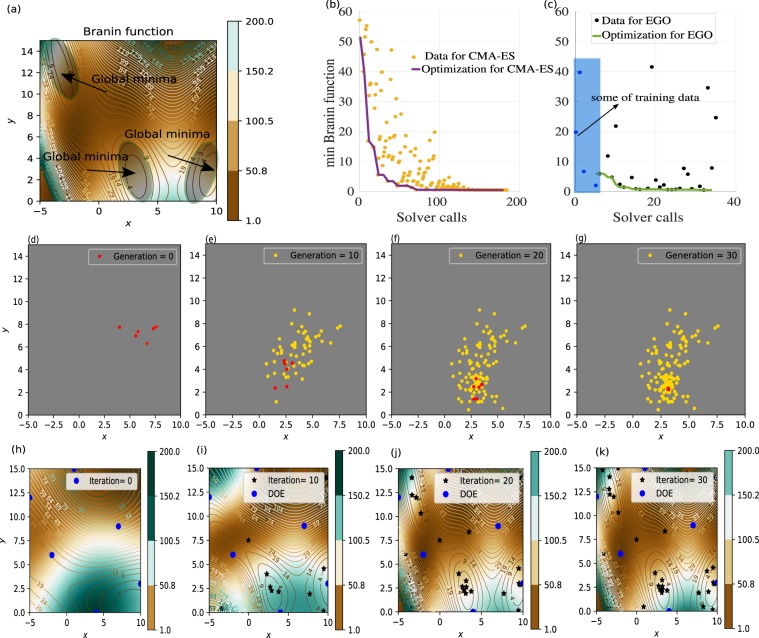
A representative 2D analytical example to illustrate the different behaviors of CMA-ES and EGO methods. The problem considered here consists in minimizing an analytical function, known as the Branin function, characterized by the presence of 3 global minima, as indicated by the black arrows in (**a**). The evolution of CMA-ES as a function of the solver calls is provided in (**b**) in which the yellow points represent the objective function values at each iteration, the purple curve indicates the best value at each iteration. (**c**) Similar to (**b**) except that here the blue points represent the DOE phase *N*_*DOE*_ = 6 (only 6 in this example). These points are used only for the initial training (blue shaded region in (**b**)) while the black dots represent the data generated during the optimization phase. The green line represents the best, optimized, results obtained during optimization phase, as explained in Fig. 1(b). (**d**–**g**) Evolution of the points tested by CMA-ES, as a function of the generation numbers (for each generation we simulate 6 designs). The yellow points represent all samples evaluated so far, the red points correspond to the last generation of size *N* = 6. This illustrates the search by progressive sampling and convergence. (**h**–**k**) Evaluation of the Gaussian process model (surrogate model) and the underlying database generated from iteration 0 to 30. Notice that the model converge to the analytical function shown in (**a**) after 30 iterations. The design points from the DOE phase are shown in blue, the black points represent the database progressively enriched during the optimization. Note that all minima are detected. As background, the GP model is plotted, which converges progressively towards the true cost function (**a**).

The Branin function *f*(*x*, *y*) depicted in Fig. 2(a) has three global minima, i.e three different combinations of parameters *x* and *y* in the range of −5 ≤ *x* ≤ 10 and 0 ≤ *y* ≤ 15 generate the same value of the objective function, as indicated by the black arrows in Fig. 2(a). Using this simple example, we highlight the different behaviors of the two algorithms. We compared the convergence and the computational efficiency of the two algorithms in Fig. 2(b,c). They depict the function value obtained with respect to the number of solver calls. As seen, both methods reach a very satisfactory function value, CMA-ES being clearly more expensive converging after about 180 solver calls, compared to 30 (including training data) for the EGO method. Below, we demonstrate the evolution of the two methods.

For CMA-ES, the yellow points shown in Fig. 2(d–g) show the evolution of all samples tested at generations ranging from 0 to 30, the red points corresponding to the last generation (size *N* = 6). CMA-ES sampling points are essentially exploring only the vicinity of the population mean, which progressively moves towards the best objective function values to converge on a local minimum.

For EGO, Fig. 2(h–k) show both the Gaussian Process model and the underlying database generated from iteration 0 to 30. At iteration 0, the database is composed of 6 points obtained from the DOE phase. Then, the database is enriched in most promising areas, yielding an improvement of the corresponding model. At iteration 30, the three regions around the global minima have been found. Moreover, a Gaussian Process model has been constructed, which is very close to the true function seen in Fig. 2(a), in which the three global points have been identified by the EGO (see black points in Fig. 2(k) and the analytical function given in Fig. 2(a).

This simple analytical example illustrate the different optimization mechanisms involved in CMA-ES and EGO algorithms. In the following, we exploit these two methods to optimize metasurface geometries for improved light deflection efficiency at *λ* = 600 *nm*.

## Numerical Results

**Figure 3:**
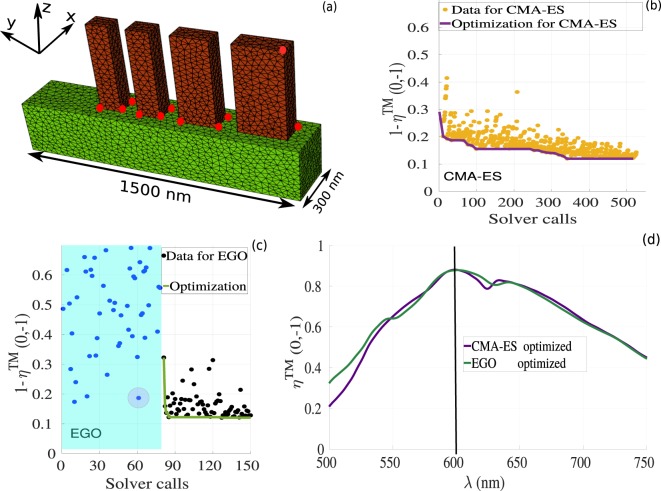
Results of the optimizations for rectangular nanoantenna arrays. (**a**) The geometry under consideration with rectangular nano ridges made of GaN (dark-red ridges) on top of a semi-infinite substrate made of Al _2_O_3_ (green region). The 12 red circles represent the optimization parameters. (**b**) Optimization process using CMA-ES as a function of the number of fullwave solver calls. Dark-yellow points represent the value of the objective function at each iteration, the solid purple line highlights the best point achieved during the optimization. In other words, for each generation we keep only the best point that minimizes the objective function. These best points that are obtained from each generation along the optimization process, are represented by the solid purple curve. (**c**) Optimization realized with the EGO solver as a function of the number of fullwave solver calls. The blue points represent the DOE (shaded region), the black points represent the value of the objective function at each optimization iteration, and the green solid line indicates the optimized data. (**d**) Comparison between the diffraction efficiency for the first order mode as a function of the wavelength for the TM polarized wave. We use purple and green colors for the CMA-ES and EGO optimized geometries, respectively; the corresponding parameter values shown in Table 1, for CMA-ES, and EGO optimized parameters.

As a first example, in Fig. 3(a), we consider a phase gradient metasurface made of rectangular GaN semi-conductor (dark-red regions) placed over a semi-infinite substrate made of Al _2_O_3_ (shown in green). We consider a normal incident plane wave with electric field polarized in the y-direction, and we aim to maximize the diffraction efficiency of the first order mode *η*(0, −1) (deflect light in the same plane of incidence y-z plane) at wavelength of *λ* = 600 *nm*. To avoid diffraction inside the substrate, we consider sub-wavelength period in the x-direction (300 *nm*) and a period of phase gradient in the y-direction, which is the dimension along which we are optimizing the geometries of subwavelength nanopillars, to be Γ = 1500 *nm*, as shown in Fig. 3(a).

In the first design, we optimize the design of rectangular antennas, considering optimization of the following parameters: the height, which is chosen equal for all pillars to comply with nanofabrication techniques; the positions of each ridges in y direction; the thicknesses in x and y directions, leading to 12 optimized parameters that are represented by the red circles in Fig. 3(a). It is worth mentioning that we took into account the experimental constraints during the optimization process, in which the minimum feature size is set to 90 *nm* and the height of the ridges is set between 600 *nm* and 800 *nm*. Figure 3(b,c) summarize the optimization results obtained for this first metasurface example. The CMA-ES results are shown in Fig. 3(b), in which the objective function evaluation at each iteration is shown in dark-yellow points, and the best values of the objective function evaluation obtained during the optimization process is represented by the purple solid curve.

**Figure 4:**
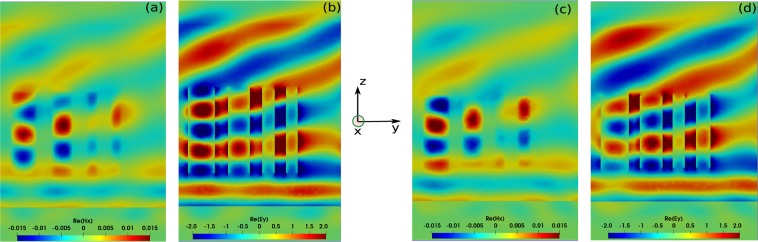
Field maps of $\Re e({H}_{x})$ and $\Re e({E}_{y})$ obtained for rectangular nanopillars for the optimized geometries at *λ* = 600 *nm*. (**a,b**) Obtained from the CMA-ES method with height *h* ≈ 800 *nm*; (**c,d**) Obtained from the EGO method with *h* ≈ 666 *nm*.

Note that the evolutionary model evaluates at each iteration exactly 11 metasurface designs, keeping the best value among the different realizations only if the latter outperform those obtained during the previous iteration (purple curve in Fig. 3(b)). After nearly 550 iterations, we obtain a global mimimum such that 1 − *η*^*TM*^(0, −1) ≈ 0.119 at *λ* = 600 *nm*, which is corresponding to diffraction efficiency of approximately 88.10% for TM polarized waves. The evolution of the diffraction efficiency *η*^*TM*^(0, −1) as a function of the wavelength is represented by the purple curve in Fig. 3(d), indicating that a maximum is achieved at *λ* = 600 *nm*. The corresponding parameter values are shown in Table 1, where *dx* and *dy* give the thicknesses in *x* and *y* directions, respectively for each rectangular element. From the result of the CMA-ES method, the optimal height of the ridges is *h* ≈ = 800 *nm*. As could be expected, a linear phase gradient, which leads to light deflection in *y*-*z* plane, is achieved by increasing the effective mode index of the nanorectangles, i.e. by gradually increasing the thicknesses in *y* direction as a function of the position of the nanoantenna within the period. Note also that the optimized height of the ridges is nearly 800 *nm*, which according to previous direct simulations and experimental works is sufficiently tall to provide high transparency windows and sufficient phase delay for increasing cross sections^12,36,37,59^. The electromagnetic field distributions for the *Re*(*H*_*x*_) and *Re*(*E*_*y*_) at *λ* = 600 *nm* are shown in Fig. 4(a,b), respectively, which clearly indicate light deflection behavior. It is worth mentioning that during the optimization process, accurate simulation results was obtained using a coarse mesh of only 3000 cells with a DGTD-${{\mathbb{P}}}_{4}$ solver (i.e. with fourth order polynomial interpolation of the components of the electromagnetic field within each mesh cell). One fullwave simulation with these mesh and solver configurations cost 26 minutes using 48 cores. A numerical convergence is provided in Fig. 8(a). The results shown in Fig. 4(a,b) have been obtained using a finer mesh and a DGTD-${{\mathbb{P}}}_{2}$ solver.

**Table 1:** CMA-ES and EGO optimized parameters for the rectangular shaped antennas shown in Fig. 3(a).

CMA-ES: element dimensions(*h* ≈ 800 *nm*)			EGO: element dimensions (*h* ≈ 666 *nm*)		
Element	*d*_*x*_(*nm*)	*d*_*y*_(*nm*)	Element	*d*_*x*_(*nm*)	*d*_*y*_(*nm*)
1	108.5	248.7	1	120.0	254.1
2	93.3	199.6	2	106.3	203.3
3	96.6	116.5	3	103.6	109.1
4	108.6	108.1	4	107.3	130.6
**CMA-ES: distance between elements**			**EGO: distance between elements**		
between (1–2)	121.4 *nm*		between (1–2)	98.5 *nm*	
between (2–3)	114.6 *nm*		between (2–3)	91.5 *nm*	
between (3–4)	103.8 *nm*		between (3–4)	101.6 *nm*	

The optimized heights obtained from CMA-ES and EGO are *h* ≈ 800 *nm* and *h* ≈ 666 *nm*, respectively.

The same optimization has also been realized using the EGO method, still considering the rectangular nanopillar setting shown in Fig. 3(a). As explained previously, this optimization approach build a DOE database in a first phase. In this example, we built a database with 80 design points represented by the blue points in Fig. 3(c). In a second phase, based on these 80 design points, a surrogate model is constructed and used during the optimization process to find a global minimum, i.e. that could achieve a better result than the actual best point found in the DOE process (represented by the pink point in Fig. 3(c)). From the green curve and its associated data (black points just above the green curve in Fig. 3(c)), one can note that the convergence is obtained after only a few number of iterations (approx. 150 iterations), beyond which the efficiency barely improves, thus indicating that the best point has been obtained. Compared to the CMA-ES, EGO minimization of the cost function optimizes the 12 parameters and converges to a global minimum in which 1 − *η*(0, −1) ≈ 0.12, corresponding to a deflection efficiency of 88.0% at *λ* = 600 *nm* after nearly 150 iterations, i.e. about 4 times faster than CMA-ES method.

Observing the optimized parameters, we realize that the geometries obtained by the EGO method (related to a specific stopping criteria) are different from those obtained with CMA-ES method (see Table 1), for the optimized parameters obtained using the CMA-ES and EGO methods), although both could provide seemingly identical diffraction efficiency of about 88% at *λ* = 600 *nm*, see Fig. 3(d).

Interestingly, the optimized parameters obtained by the EGO method are going against the intuitive designs for which linear phase gradients rely on increasing effective mode index of the antennas as a function of the pillar transverse section. Indeed, the expected gradient in the *d*_*y*_ thicknesses of the ridges is not fully satisfied as the *d*_*y*_ of the last ridge is larger than the previous pillar. These results are sensibly different to those obtained using the CMA-ES method. Note also that the global thicknesses of the ridges in *y* direction are slightly thicker than their counterparts obtained using the CMA-ES method. In addition, the height of the nanoridges is also much shorter than the optimized height found by the CMA-ES method by about nearly one wavelength. Similarly to the analytical example shown in Fig. 2, the noticeable differences in the parameters indicates that EGO has reached another global optimum, which was not found by the CMA-ES method. The difference in structural parameters is also reflected in the field maps provided in Fig. 4, in which we clearly see that the number of longitudinal modes for the optimized geometry found by the EGO method shown in Fig. 4(a,b) are different from the ones obtained by the CMA-ES method shown in Fig. 4(c,d). The possibility of operating in the optimal transmission regime, also related to the Kerker condition in Mie theory, has been discussed recently. Since the height of nanopillar controls essentially the longitudinal resonance, similar light deflection response can be achieved by varying the transverse cross sections of nanopillar arrays having two distinct heights, thus explaining why we could achieve at least two different global mimima in this configuration. We emphasize that in our unit cell, which is made of 4 nanoridges, optimization is performed by modifying all antennas parameters at once, thus properly optimizing the near-field coupling between the different modes, as illustrated in Fig. 4.

**Figure 5:**
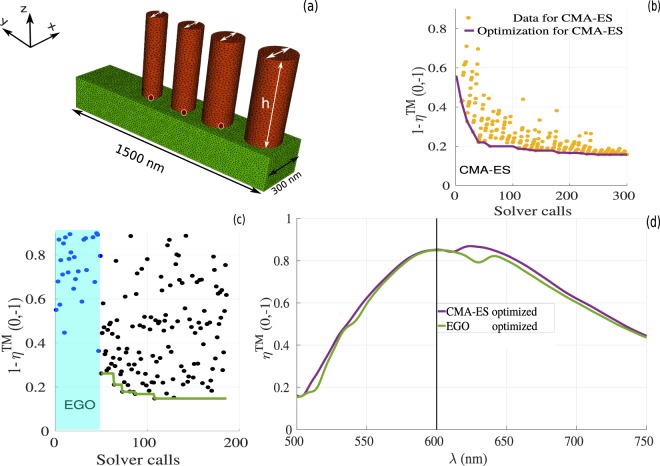
Optimization results for cylindrical nanopillars arrays. Similarly to Fig. 3, (**a**) is the geometry under consideration with cylindrical nanoridges made of GaN (dark-red ridges) on top of a semi-infinite substrate made of Al _2_O_3_ (green region). The 8 red circles represent the optimization parameters. (**b**) Optimization process using CMA-ES as a function of the fullwave solver calls. Dark-yellow points represent the value of the objective function at each iteration, the solid purple line indicates the best point during the optimization up to the current iteration. (**c**) Optimization using the EGO method as a function of the fullwave solver calls. The blue points represent the DOE (shaded region), the black points represent the value of the objective function at each optimization iteration, and the green solid line indicates the optimized data. (**d**) Comparison between the diffraction efficiency for the first order mode as a function of the wavelength for the TM polarized wave. Purple and green colors for the CMA-ES and EGO optimized geometries, respectively; the corresponding parameter values shown in Table 2, for the CMA-ES and EGO results. The field maps for $\Re e({H}_{x})$ and $\Re e({E}_{y})$ for the optimized geometries at *λ* = 600 *nm* are shown in Fig. 6.

For the second example, we optimize a phase gradient metasurface made of cylindrical nanopillars (see Fig. 5(a)) to maximize the diffraction efficiency in the first order mode. Due to the symmetry of the nanopillars, we are targeting polarization insensitive properties. For this example, we optimized the structures by modifying 8 parameters (see Fig. 5(a)) including the pillars diameter (thick white arrows), the height, and the position of the nanopillars as indicated by the red points. We also kept experimental constraints identical to those for the rectangular case. We begin by analyzing again the optimization of the CMA-ES case, as shown in Fig. 5(b), for which a global point with diffraction efficiency around 85% for TM polarized waves (results for TE polarization is also around 85%, data not shown) is obtained after 300 fullwave solver calls. For the EGO method, we consider a DOE database with 50 design points (see blue points in Fig. 5(c)). Then, a surrogate model is constructed and a global optimum is clearly obtained after nearly 180 iterations with efficiency 85%. For this example, both optimization methods lead to structures with increasing gradient in the diameter, but the two set of parameters obtained by CMA-ES and EGO are slightly different as indicated in Table 2. The diffraction efficiency as a function of the wavelength obtained by the two methods can be found in Fig. 5(d), where we clearly see slight differences between the results obtained by the CMA-ES (purple curve) and the EGO (green curve) method.

**Table 2:** CMA-ES and EGO optimized parameters for the cylindrical nanopillars shown in Fig. 5(a).

CMA-ES: element dimensions(*h* ≈ 735 *nm*)		EGO: element dimensions (*h* ≈ 705 *nm*)	
Element	Diameter (*nm*)	Element	Diameter (*nm*)
1	209.7	1	210.0
2	176.2	2	189.0
3	149.1	3	154.5
4	117.6	4	126.0
**CMA-ES: distance between elements (center to center)**		**EGO: distance between elements (center to center)**	
between (1–2)	308.6 *nm*	between (1–2)	293.9 *nm*
between (2–3)	255.7 *nm*	between (2–3)	279.4 *nm*
between (3–4)	249.3 *nm*	between (3–4)	236.9 *nm*

The optimized heights obtained from CMA-ES and EGO are *h* ≈ 735 *nm* and *h* ≈ 708 *nm*, respectively.

**Figure 6:**
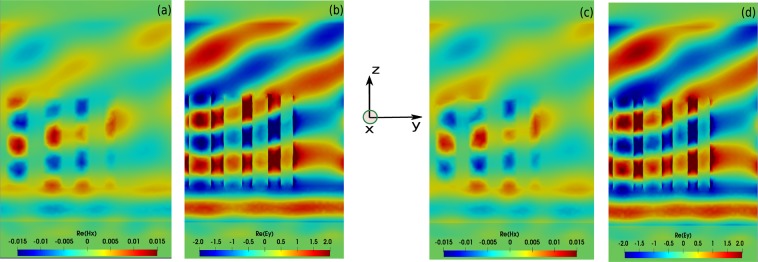
Field maps of $\Re e({H}_{x})$ and $\Re e({E}_{y})$ obtained for cylindrical nanopillars for the optimized geometries at *λ* = 600 *nm*. (**a,b**) Obtained from the CMA-ES method with height *h* ≈ 735 *nm*, (**c,d**) Obtained from the EGO method with *h* ≈ 708 *nm*.

The DGTD method allows us to obtain the wavelength dependence of the deflection efficiency for both CMA-ES and EGO, essentially showing similar behavior. We denote a slight difference, which can be attributed to the fact that both structures are not operating on the exact same longitudinal modes, see the field maps in Fig. 6 obtained at *λ* = 600 *nm*. The CMA-ES optimum is achieved for *h* ≈ 735 *nm*, for which nearly half of the last mode field lobe is located in the first ridge, while EGO optimization results gives an optimal height *h* ≈ 708 *nm*.

**Figure 7:**
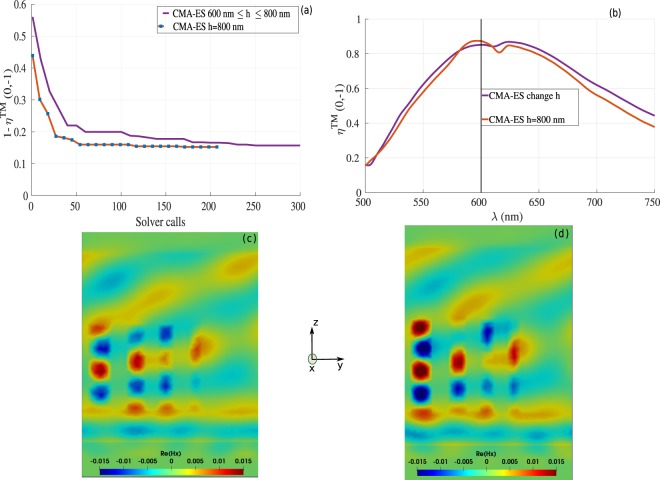
(**a**) Comparison between results obtained with CMA-ES (for the cylindrical nanopillars) as a function of height (purple curve, exactly as in Fig. 5(b) in which the height is optimized to *h* ≈ 735 *nm*), and the ones obtained with CMA-ES with fixed *h* = 800 *nm* (dark orange curve) as a function of the number of fullwave solver calls. The corresponding optimized parameter values for the case with *h* = 800 *nm* (dark orange curve) can be found in Table 3. (**b**) Diffraction efficiency as a function of the wavelength. (**c**,**d**) Field maps of $\Re e({H}_{x})$ at *λ* = 600 *nm* for the reference case with *h* ≈ 735 *nm* (purple curve in (**a**)), and the case with *h* = 800 *nm* (dark orange curve in (**a**)).

**Figure 8:**
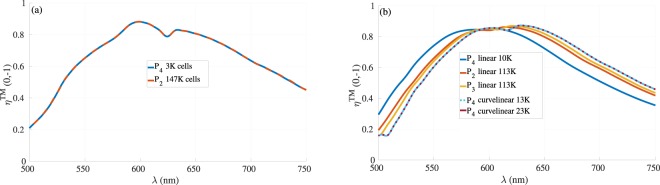
Convergence study using the DGTD method (**a**): for the rectangular shaped antenna (for CMA-ES results, the corresponding parameters can be found in Table 1) using coarse mesh with 3000 cells with fourth order polynomial *P*_4_ order (blue curve) and with finer mesh size (147 000 cells) with second order polynomial *P*_2_ order (purple curve). (**b**): convergence results for the cylindrical shaped antenna using the data shown in Table 2 for the CMA-ES results using different mesh sizes and/or types, different polynomial orders.

**Figure 9:**
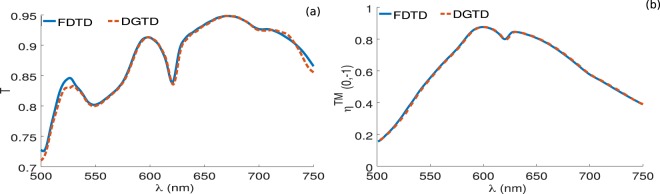
Comparison between results obtained with the FDTD method (blue solid curves) and the ones obtained with the DGTD method (orange dashed curves) for the optimized cylindrical nanopillars shown in Table 3. (**a**) For the total transmission and (**b**) for the deflection efficiency for the first order mode. The FDTD results are obtained with nearly 10^5^ cells, however in our DGTD solver, we used only 13 000 cells with higher order polynomial order and curvilinear elements.

To capture the influence of the height on the performance of the designed metasurfaces, we decided to compare the CMA-ES results for a fixed height *h* = 800 *nm* with those obtained when including the height in the set of optimization parameters, see Fig. 6(b)). The comparison in Fig. 7(a) clearly illustrates that the results obtained with a fixed height *h* = 800 *nm* (dark orange curve) approach those obtained with varying the height, including the set of parameters obtained from both structures that converge to each other, see Tables 2 and 3 for the reference case with *h* ≈ 735 *nm* and the fixed height case *h* = 800 *nm*, respectively. This indicates that the maximum of efficiency found in this configuration is quite robust, being resilient to significant height variation. To properly account for uncertainties due to the meshing of the nanostructures, we have realized a convergence proof considering the cylindrical nanopillar array, see Fig. 8(b), in which we investigated different mesh sizes and different polynomial orders. It is worth mentioning that during the optimization process, we used a coarse mesh of 10 000 cells with fourth order polynomial ${{\mathbb{P}}}_{4}$, which proved sufficient to get accurate results (one fullwave simulation with this mesh and interpolation order specifications takes about 40 minutes using 48 cores), as it is depicted in Fig. 8(b). To present accurate field distributions and improve the visualization, the results obtained for the optimized geometries shown in Figs. 6(a,d) and 7 have been obtained with a finer mesh and a DGTD-${{\mathbb{P}}}_{2}$ solver. As shown in Figs. 8(b) and 9, it is possible to further improve the numerical accuracy using higher order curvilinear elements in combination with higher order polynomials. However, current experimental realization of metasurfaces using state of the art nanofabrication facilities do not reach such fine level of details and it is therefore not essential to further improve the nanostructure discretization to even higher numerical resolution. At this point, it is instead more interesting to point out that after various attempts to achieve close to unitary deflection efficiency, all optimized structures converge towards roughly the same number of 0.85.

**Table 3:** CMA-ES optimized parameters for the cylindrical nanopillars shown in Fig. 5(a) with fixed height *h* = 800 *nm* during the optimization.

CMA-ES: element dimensions		CMA-ES: distance between elements (center to center)	
Element	Diameter (*nm*)		
1	205.1	between (1–2)	328.0 *nm*
2	176.4	between (2–3)	264.3 *nm*
3	142.3	between (3–4)	238.5 *nm*
4	128.2

This limitation of the deflection efficiency is expected as demonstrated in refs. ^60,61^. According to these works, the efficiency of the phase gradient metasurfaces is limited due to impedance mismatching between the incident and the desired wavefront, leading to an increase of the scattering in the other modes. In particular, theoretical proofs have shown that a unitary light deflection to an arbitrary angle cannot be achieved using linear gradient phase profiles^60^.

This effect is sufficiently important to avoid full unitary efficiency^60^. In order to overcome this limitations and achieve a unitary efficiency for light deflection, two different approaches have been proposed. The first approach is based on designing a metasurface with a surface impedance profile that takes into account the impedance matching into consideration. A balance between gain and loss elements (active metasurfaces), or nonlocal effects must be considered. This first approach is relatively difficult to be considered from the fabrication point of view, it requires complex design fabrication, and deep sub-wavelength resolution. Another technique is introduced in ref. ^61^. In this work, the authors have shown that by using a lossless bi-anisotropic resonators with coupled magnetic and electric field responses located above a ground plane (the distance between the resonators and the ground plane must be optimized in order to achieve the maximum efficiency). Even if the authors focused on the reflective case with no losses (ideal case), they mention that this work can be extended to the case of transmitting metasurfaces, by considering a two-layer metasurface. This latest technique requires using resonators with strong magnetic and electric responses at visible frequencies.

Besides the fact that we observed a limitation in the overall efficiency, as expected from previous theoretical predictions^60,61^, these studies for cylindrical and rectangular nanoantennas allow us to confirm that the optimization strategies based on the CMA-ES and EGO methods that we have considered here are capable of *understanding* which key parameter influence the most on the minimization process. Indeed, for all the results presented herein, we figured out that both optimizers set the thickness of the first ridge (*d*_*y*_ in case of rectangular nanopillars, and diameter in case of cylindrical nanopillars) to the largest values, for which the effective refractive index is the largest. The height of the ridges is another interesting parameters as it essentially relates to the different longitudinal modes inside the nanopillars, leading to relatively well optimized diffraction efficiency at different height.

## Conclusion

Two advanced optimization strategies have been introduced to optimize 3D semiconductor-based metasurfaces. We proved that our optimization methods are very efficient in obtaining different global minima/maxima, requiring only a few hundreds of electromagnetic solver calls. We applied these methods to maximize the light deflection efficiency at *λ* = 600 *nm* using GaN based metasurfaces. Our numerical results reveal that one can obtain 85% of deflection efficiency for both TM and TE polarization using cylindrical nanoantennas. In addition, we show that using rectangular nanoantennas, one might obtain more that 88% of deflection efficiency for TM polarization. These methods, which are widely used in the computational fluid dynamics community, could have significant implications in the design of efficient metasurfaces, notably in view of their utilization in real world applications, for which reaching highly efficient designs in reasonable computation time is one of the most important figure of merit. Our future works will aim at improving the computational performances of these optimization strategies by leveraging the different levels of parallelism underlying their algorithmic structures one one hand, and extending their capabilities in view of dealing with multi-objective problems.

### Numerical validation

In this section, we discuss about the numerical validation of some of the results obtained in our work. First, we start by studying the influence of the mesh size/type using our DGTD fullwave solver from the DIOGENeS software suite^41^ on some of the optimized designs. In Fig. 8(a), we study the influence of the mesh and the polynomial order on the optimized solution obtained in the rectangular nanoantennas case (the results obtained from the CMA-ES method are shown in Fig. 3). As it can be seen in Fig. 8(a), using fourth order polynomial order ${{\mathbb{P}}}_{4}$ with a coarse mesh (only 3 000 cells) provides the same results than when we consider second order polynomial order ${{\mathbb{P}}}_{2}$ with a finer mesh (with 147 000 cells). In Fig. 8(b), we show the convergence for the cylindrical nanoantennas case obtained using the CMA-ES method with *h* ≈ 735 *nm* (the corresponding parameters can be found in Table 2) using both linear tetrahedral elements and also curvilinear tetrahedral elements in order to achieve a high order approximation of the cylindrical geometry of the nanopillars. As it can be noticed, the convergence is obtained with less cells in the case of the mesh with curvilinear elements compared to the case with linear ones. To conclude on this point, using our DGTD solver, we are able to demonstrate the convergence for the optimized geometries using different mesh size and/or types, which is not trivial especially for the case of cylindrical elements that require higher order curvilinear mesh type for more accurate results, otherwise, a finer mesh must be used with the classical linear elements.

In Fig. 9, we provide another validation of our numerical results by simulating the optimized structure given in Table 3 using the FDTD fullwave solver from Lumerical commercial software, and comparing the results with the ones obtained from our DGTD solver. As it can be seen from Fig. 9, we have a very good agreement between the results obtained using the two methods for both the total transmission and the deflection efficiency of the first order mode. However, in the case of the DGTD method, we show the results using curvilinear elements with fourth order polynomial using only 13 000 cells (same results can be obtained with classical linear elements with 109000 cells as we have seen in Fig. 7). On the other hand, for the FDTD results, we consider a very fine mesh with nearly 10^5^ cells in order to get accurate results that can be compared with the DGTD method.

**Figure 10:**
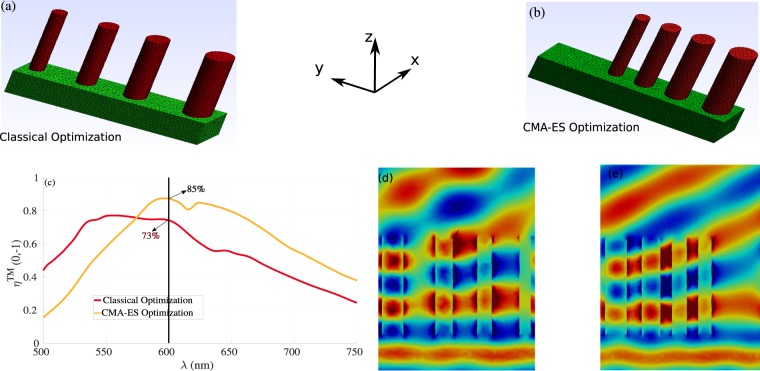
Comparison between the classical approach to phase gradient metasurface design and our optimized geometries for the cylindrical nanopillars with *h* = 800 *nm*. (**a**,**b**) The geometry obtained using the classical approach in which each nanopillar is optimized manually by changing the diameter and finally placed together in order to obtain the desired phase shift needed to maximize the light deflection for the first order mode at *λ* = 600 *nm* with period 1500 *nm* in *y*-direction. (**c**) Results obtained using the CMA-ES for the cylindrical nanopillars (see Table 3) for the corresponding parameters. (**c**) Comparison between the deflection efficiency for the first order mode obtained using the classical (red curve) and the CMA-ES (orange curve). (**d**,**e**) Represent field maps of $\Re e({E}_{y})$ obtained using the classical optimization design and the CMA-ES results, respectively.

Next, we show one example in order to demonstrate that our optimization techniques outperform the classical approach to phase gradient metasurface design. In Fig. 10(a), we present the geometry obtained using the classical approach. In this approach, one calculates the phase gradient needed with 1500 nm period in *y*-direction at wavelength *λ* = 600 *nm* and place the pillars at the right *y* positions in order to introduce the needed phase shifts to maximize the light deflection of the (0, −1) mode. The phase shifts and transmission introduced by each single element is calculated before using a classical forward simulation by changing the radius of the pillar and compute the corresponding phase and transmission (we consider a fixed height *h* = 800 *nm*). The corresponding deflection efficiency for the first order mode can be seen in the red curve in Fig. 10(c), in which the maximum efficiency at *λ* = 600 *nm* is around 74%. On the other hand the optimized geometry obtained using the CMA-ES method presented in Fig. 10(b), provides nearly 85% of deflection efficiency at *λ* = 600 *nm* (data can be found in Table 3). This discrepancy between the two results is clearly linked to the near field coupling as it is shown in Fig. 10(d,e). In the classical design approach (see the corresponding geometry in Fig. 9(a)), we neglect the effect of the near field coupling when we place the nanopillars together to construct the required phase shift, while in the optimization techniques presented in this paper, the near field coupling is taking into account during all optimization steps, which give us an optimized geometry as shown in Fig. 10(b).
